# The Potential Role of Proinflammatory Cytokines and Complement Components in the Development of Drug-Induced Neuropathy in Patients with Multiple Myeloma

**DOI:** 10.3390/jcm10194584

**Published:** 2021-10-04

**Authors:** Karolina Łuczkowska, Magdalena Rutka, Dorota Rogińska, Edyta Paczkowska, Bartłomiej Baumert, Sławomir Milczarek, Martyna Górska, Piotr Kulig, Bogumiła Osękowska, Michał Janowski, Krzysztof Safranow, Krzysztof Sommerfeld, Ewa Borowiecka, Piotr Zawodny, Anna Koclęga, Grzegorz Helbig, Bogusław Machaliński

**Affiliations:** 1 Department of General Pathology, Pomeranian Medical University, 70-111 Szczecin, Poland; karolina.luczkowska@pum.edu.pl (K.Ł.); magdalena.gibka@pum.edu.pl (M.R.); doroginska@gmail.com (D.R.); edyta.paczkowska@pum.edu.pl (E.P.); slawek.milczarek@gmail.com (S.M.); martgors@gmail.com (M.G.); piotrkulig@interia.eu (P.K.); estetic@estetic.pl (P.Z.); 2 Department of Bone Marrow Transplantation, Pomeranian Medical University, 71-252 Szczecin, Poland; bbaumert@pum.edu.pl (B.B.); bogumilaosekowska@gmail.com (B.O.); janowskimm@gmail.com (M.J.); krzysztof.sommerfeld@gmail.com (K.S.); eborowiecka@poczta.fm (E.B.); 3 Department of Biochemistry and Medical Chemistry, Pomeranian Medical University, 70-111 Szczecin, Poland; chrissaf@mp.pl; 4 Department of Hematology and Bone Marrow Transplantation, Medical University of Silesia, 40-027 Katowice, Poland; annakkoc@wp.pl (A.K.); ghelbig@o2.pl (G.H.)

**Keywords:** peripheral neuropathy, drug-induced peripheral neuropathy, multiple myeloma, proinflammatory factors

## Abstract

The launch of novel chemotherapeutic agents—in particular, proteasome inhibitors and immunomodulatory drugs—dramatically changed multiple myeloma (MM) therapy, improving the response rate and prolonging progression-free survival. However, none of the anti-MM drugs are deprived of side effects. Peripheral neuropathy (PN) seems to be one of the most pressing problems. Despite extensive research in this area, the pathogenesis of drug-induced peripheral neuropathy (DiPN) has not yet been fully elucidated. In the present study, we aimed to assess the potential relationship between proinflammatory factors and the development of PN in MM patients with particular emphasis on the application of VTD (bortezomib, thalidomide, dexamethasone) regimen. Our analysis identified increased concentrations of CCL2, IL-1β, and IFN-γ in plasma of MM patients during treatment, both with and without symptoms of PN, compared with untreated neuropathy-free MM patients. At the same time, the plasma concentration of IL-1β in patients with neuropathy was significantly increased compared with patients without PN before and during treatment. Moreover, the results were enhanced at the transcript level by performing global mRNA expression analysis using microarray technology. The most significant changes were observed in the expression of genes responsible for regulating immunological and apoptotic processes. An in-depth understanding of the mechanisms responsible for the development of DiPN might in the future reduce the incidence of PN and accelerate diagnosis, allowing the choice of neuropathy-free treatment strategies for MM.

## 1. Introduction

Multiple myeloma (MM) is a plasma cell malignancy characterized by bone marrow infiltration by clonal plasma cells and typically accompanied by the presence of monoclonal immunoglobulins in blood or urine. It is commonly believed that MM is preceded by a premalignant state—monoclonal gammopathy of undetermined significance (MGUS) [1], which transforms to MM at a rate of 1% per year [2]. The pathogenesis of MM is still not entirely elucidated, yet there are multiple known mechanisms underlying myelomagenesis. There are several oncogenes that are thought to be of a paramount importance. Cyclin D is a cell cycle regulator that promotes G1/S transition and is encoded by the CCND1 gene. It is suggested that dysregulation of cyclin D is a unifying factor in MM pathogenesis [3]. Moreover, cyclin D is thought to generate oxidative stress in myeloma cell and, therefore, increase cell migration and adhesion. This phenomenon is peculiar to MM cells expressing cyclin D1 [4]. In addition, Chinen et al. described a novel exon CCND1.tv. that may serve for diagnosis and minimal residual disease (MRD) assessment in mantle cell lymphoma (MCL) and MM with chromosome 11q13 abnormalities [5]. Transcription factor NF-κB controls various biological processes and contributes to the development of many malignant neoplasms [6], including MM. In MM, transcription factor NF-κB regulates the expression of genes encoding factors such as IL-6, BAFF, and MIP-1α, which contribute to the growth of malignant plasma cells and the development of the disease [7]. There is a strong correlation between MYC expression and translation activity in MM and other hematological malignancies [8]. Research conducted in recent years has shown that epigenetic mechanisms also contribute to the pathogenesis of MM [9]. Fernández de Larrea et al. demonstrated a correlation between the grade of total DNA methylation and survival time in patients with relapsed MM treated with bortezomib (BTZ). Patients with total DNA methylation higher than 3.95% reached a longer overall survival time. In contrast, patients with a relatively low percentage of total methylation (<1.07%) of the NFKB1 gene showed longer overall survival after BTZ-therapy [10].

Peripheral neuropathy (PN) is common among MM patients and is related to either the disease itself or its treatment. Drug-induced PN (DiPN) occurs relatively frequently in patients and depends on the treatment schedule, the dose used, and the method of administration [11]. It occurs in approximately 40% of patients under treatment with the bortezomib-containing regimen [12], and in up to 70% of patients on thalidomide [13]. Many different scales are used to classify the degree and type of neuropathy, but the most common is the National Cancer Institute Common Terminology Criteria for Adverse Events (NCI-CTCAE) [14]. This scale includes three types of neuropathy (sensor neuropathy, sensor-autonomy, sensorimotor) and 4 grades (where 0 is no symptoms and 4 is permanent functional impairment) [15]. Although the etiology of neuropathy is largely unknown, there are several mechanisms such as amyloid deposition, immunoglobulin M antibodies directed at myelin-associated glycoprotein, a glycoconjugate component of nerves involved in interactions between Schwann cells and axons, and cytokine-mediated injury that presumably contribute to the development of MM-related PN [16]. There is evidence that several cytokines, including TNF-α, IL-6, TGF-β, and IL-1β, may participate in bortezomib-induced PN [17,18]. In addition, coadministration of anti-TNF-α in BTZ therapy may be a promising strategy to prevent the development of PN [18]. Nonetheless, Liu et al. stated that inhibition of TRPA1 and IL-6 signal might alleviate symptoms of BTZ-induced PN [19].

Aforementioned studies suggest a crucial role of inflammation and proinflammatory cytokines in chemotherapy-induced PN among MM patients.

To better understand the complex pathogenesis of DiPN development with a particular emphasis on the VTD (bortezomib, thalidomide, dexamethasone) regimen, in the present study, we examined global mRNA expression and the concentration of both proinflammatory factors and complementary components in the plasma of MM patients.

## 2. Materials and Methods

### 2.1. Subjects and Initial Management

120 patients with MM were recruited from the Department of Bone Marrow Transplantation of the Pomeranian Medical University in Szczecin and the Department of Hematology and Bone Marrow Transplantation, Medical University of Silesia in Katowice. None of the recruited patients had been diagnosed with PN in the course of MM itself. All patients enrolled in the study signed an informed consent in accordance with the tenets of the Declaration of Helsinki.

PN was assessed and classified in patients with MM during diagnosis and treatment based on medical history, and physical examinations were conducted according to the International Myeloma Working Group (IMWG) guidelines using the National Cancer Institute Common Terminology Criteria for Adverse Events (NCI-CTCAE) scale. On this basis, patients were recruited into 3 subgroups: I) previously untreated MM patients without symptoms of PN (*n* = 59); II) treated MM patients with symptoms of PN (*n* = 34); III) treated MM patients without symptoms of PN (*n* = 27).

### 2.2. Material

Peripheral blood samples were collected once prior to qualification for the introduction of the chemotherapy (previously untreated group without PN) and once prior to the next treatment cycle (both treated groups with or without PN). Minimum discontinuation of bortezomib and lenalidomide prior to blood testing was 10 days and 8 days, respectively. Peripheral blood samples (~5 mL) collected in EDTA tubes were centrifuged (2000 rpm, 10 min); then, the plasma was collected in a new tube and centrifuged again under the same conditions. The plasma samples were stored at −80 °C. Plasma-free blood was lysed with Lysing Solution (BD Biosciences, San Jose, CA, USA) to obtain blood nucleated cells. 

Peripheral blood mononuclear cells (PBMCs) were selected for the study due to the easy, noninvasive, and quick method of collecting this biological material, which is extremely important in the search for biomarkers. Despite the heterogeneity of PBMCs, they are often successfully used to analyze gene expression as a predictive biomarker [20,21,22]. The most important feature of a good biomarker is its high specificity and sensitivity characteristic for the selected disease, regardless of the biological material used.

### 2.3. RNA Isolation

Total RNA was isolated from blood mononuclear cells using the commercial mirVana miRNA Isolation Kit (Thermo Fisher Scientific, Waltham, MA, USA). Isolation was performed according to the manufacturer’s protocol. The final concentration and quality of total RNA isolated from cells was determined by the Epoch spectrophotometer (Biotek, Winooski, VT, USA).

### 2.4. Affymetrix GeneChip Microarray and Data Analysis

mRNA microarrays were executed only on representative samples to identify specific genes and processes important in the development of neuropathy. In each group, 5 random samples were selected and combined, and genetic array analysis was performed on 3 technical replicates. In the next stage of the study, the expression of selected genes was confirmed in all patients. Affymetrix Human Gene 2.1 ST Array Strips (Affymetrix, Santa Clara, CA, USA) were used for the study and performed in *n* = 3 technical replicates. Microarrays were made and analyzed according to the methods described in the previous articles [23,24]. In addition to the genetic arrays analysis presented in the article, we performed clusterProfiler and GSEA analysis (data not shown).

### 2.5. Validation of Data Obtained from Microarrays

Primers for gene validation were designed by BLAST PRIMER and purchased from the Laboratory of DNA Sequencing and Oligonucleotide Synthesis, Institute of Biochemistry and Biophysics, Polish Academy of Sciences, Warsaw, Poland. The qRT-PCR program consisted of 4 steps: 10-min initial denaturation at 95 °C, denaturation at 95 °C for 15 s, annealing at 60 °C for 5 s, and extension at temperature depending on the selected primer for 10 s. The relative gene expression was quantified using the comparative Ct method 2ΔCt. BMG was set as a reference gene. All products were characterized by high specificity, which was checked by determining melting points (0.1 °C/s transition rate).

After reverse transcription using the First Strand cDNA Synthesis Kit (Thermo Fisher Scientific, Waltham, MA, USA) was performed, the qRT-PCR reaction mixture (10 μL) contained 5 μL of SYBR Green PCR Master Mix (Bio-Rad, Hercules, CA, USA), 1 μL cDNA template, 1.2 μL specific primers (0.6 μL reverse primer and 0.6 μL forward primer), and 2.8 μL Nuclease-Free Water. Gene expression studies were performed on the Bio-Rad CFX96 Real-Time PCR Detection System (Bio-Rad Inc., Hercules, CA, USA).

### 2.6. Luminex Analysis

The Luminex method allows the simultaneous measurement of many factors in the same sample. The basis of this method is the use of magnetic microspheres marked inside with a fluorescent dye, which is an individual spectral address of the microsphere. Moreover, the microspheres are coated with antigens or antibodies on the surface, depending on the factor we want to measure. In this study, commercial kits Luminex Human Discovery Assay (R&D Systems, Minneapolis, MN, USA) were used to measure the concentration of TNF-α, IL-6, Chitinase3, IL-10, CCL2, IL-1β, and IFN-γ. The first step of the reaction was the incubation of the microspheres (50 µL/well) with the plasma of patients (50 µL/well) from all groups at room temperature (RT) on horizontal orbital microplate shaker (800 rpm) in the dark. After 2 h, the wells were washed with 100 µL of wash buffer (WB) 3 times using a handheld magnet. Then, the detection-biotin-antibody cocktail (50 µL/well) was added to the plate and incubated for 1 h under the same conditions as in the first step. In the last step, Streptavidin–PE was added and incubated for 30 min under the same conditions as in the first step. The measurement was carried out on a Luminex 200 apparatus (Luminex Corporation, Austin, TX, USA). The concentrations were converted according to a standard curve consisting of 6 points.

### 2.7. Statistical Analysis

The Mann–Whitney test was used in the analyses to compare the quantitative parameters between the groups. Spearman’s rank correlation coefficient (rs) was used to measure the strength of the relationships between the levels of mRNA expression, the concentration of proinflammatory factors, and quantitative clinical parameters. Fisher’s exact test was used to compare the qualitative parameters between the groups. A *p* value of <0.05 was considered statistically significant. Statistica 13 software (Dell Inc., Oklahoma City, OK, USA) was used for statistical analyses.

## 3. Results

### 3.1. Study Group Characteristics

The patients enrolled in the study were divided into three groups: (i) patients with MM before treatment without neuropathy; (ii) patients with MM who developed symptoms of neuropathy during treatment; (iii) patients with MM who did not develop symptoms of neuropathy during treatment. Patients were treated with various regimens, but the most common was VTD (bortezomib, thalidomide, dexamethasone; 60%). The other treatment regimens included the following: VMP (bortezomib, melphalan, prednisone; 13.33%); RD (lenalidomide, dexamethasone; 13.33%) (prior to treatment with RD, patients always received a bortezomib-containing regimen, of which 44.4% of patients were previously treated with VTD scheme, 33.3% with VD, and 11% with VCD and VMP); VD (bortezomib, dexamethasone; 6.66%); VCD (bortezomib, cyclophosphamide, dexamethasone; 5%); VRD (lenalidomide, bortezomib, dexamethasone; 1.66%). The study group with PN included only patients who were ineligible for AHSCT or prior to the AHSCT procedure. In the non-PN treatment group, there were 4 previously melphalan-conditioned patients supported with AHSCT.

DiPN was determined according to the NCI-CTCAE scale. Most often, the patients showed symptoms of sensory neuropathy (66.66%); 16.66% of patients had sensorimotor and 16.66% had sensory-autonomic neuropathy. An analysis of all types of neuropathies showed that 15.16% of patients had grade 1 neuropathy, 60.6% grade 2, and 24.24% grade 3. Symptoms of neuropathy appeared at the earliest, after the 1st cycle of treatment with the VTD regimen, and at the latest, after the 23rd cycle of treatment with the RD regimen. Considering only the VTD regimen, which was the most frequently used scheme, the minimum number of cycles to develop neuropathy was 1 and the maximum number was 6 cycles. Furthermore, we have assessed the mean cumulative bortezomib dose at sampling time in the PN group (23.68 ± 5.54 mg/m^2^) and in patients without PN during treatment (30.92 ± 14.65 mg/m^2^). The differences were statistically insignificant (*p* = 0.08, *t*-test). However, the trend towards higher cumulative doses of bortezomib used in the group of patients without PN during treatment probably resulted in a significantly lower number of leukocytes in this group (Table 1). In the PN group during treatment, there were 30% more patients who received thalidomide (VTD, 100 mg/day a la longue) in their regimen compared with patients without PN. Similarly, the lenalidomide regimen (Rd and VRD, 25 mg/day for three weeks and one week off) was received by 18% more patients in the PN group than in the non-PN group during treatment.

It should be emphasized that the presence of PN in patients with multiple myeloma may result from the disease itself (approximately up to 20%) or the presence of other predisposing factors, such as diabetes mellitus (DM), alcohol abuse, vitamin B12 and folic acid deficiency, and viral infections. That is why we evaluated the study group in terms of the abovementioned factors. None of the patients with MM had PN before initiation of treatment. Among patients with PN (*n* = 34), 1 patient was diagnosed with DM (oral treatment) and 1 patient was HBc (+) with a negative HBV-DNA PCR test at the same time. None of the patients with PN had alcohol abuse, HCV infection, or vitamin B12 and folic acid deficiency. Among patients without PN (before and during treatment *n* = 86), 3 were diagnosed with DM (oral treatment), 1 abused alcohol, 2 were HBc (+) with simultaneous negative HBV-DNA PCR results, 1 was vitamin B12 deficient, and 6 were folic acid deficient. A detailed statistical analysis including treatment, clinical parameters, and examined factors was performed (Table 1).

### 3.2. Gene Expression Profile in MM Patients

#### 3.2.1. Microarray Analysis

**Figure 1:**
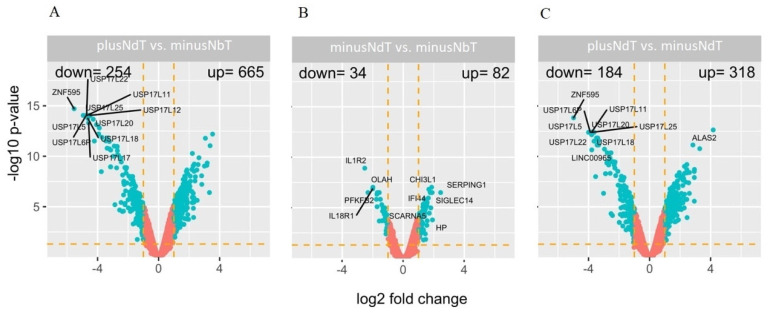
The volcano plot of global gene expression. Graphs (**A**–**C**) show comparisons with different groups. (**A**) patients with neuropathy during treatment (plusNdT) vs. the group of patients without neuropathy before treatment (minusNbT); (**B**) patients without neuropathy during treatment (minusNdT) vs. the group of patients without neuropathy before treatment; (**C**) patients with neuropathy during treatment vs. patients without neuropathy during treatment. Genes are represented by dots. The graphs show the genes with at least two-fold change and *p* < 0.05. The graphs also contain the symbols of the genes with the largest change in expression.

Bioinformatics analysis of genetic microarrays showed decreased the expression of 254 genes at least two-fold (log2_fold change −2 to −46.52, *p* < 0.05) and the expression of 665 genes increased at least two-fold (log2_fold change 2 to 11.60, *p* < 0.05) in samples of myeloma patients with neuropathy during treatment (plusNdT) compared with the group of patients with MM without neuropathy before treatment (minusNbT) (Figure 1A). On the other hand, the comparison of the group of patients with MM without neuropathy during treatment (minusNdT) vs. the group of patients with minusNbT, revealed the reduced expression of 34 genes at least two-fold (log2_fold change −2 to −5.76, *p* < 0.05) and increased expression of 82 genes at least two-fold (log2_fold change 2 to 5.45, *p* < 0.05) (Figure 1B). Moreover, a comparative analysis of the group with neuropathy during treatment was performed in relation to patients without symptoms of neuropathy during treatment. The obtained results showed that 184 genes were downregulated at least two-fold (log2_fold change −2 to −31.57, *p* < 0.05) and 318 genes were upregulated at least two-fold (log2_fold change 2 to 18.01, *p* < 0.05) (Figure 1C). Detailed fold values of selected up- or downregulated genes are presented in Supplementary Table S1. 

#### 3.2.2. DAVID Analysis

**Figure 2:**
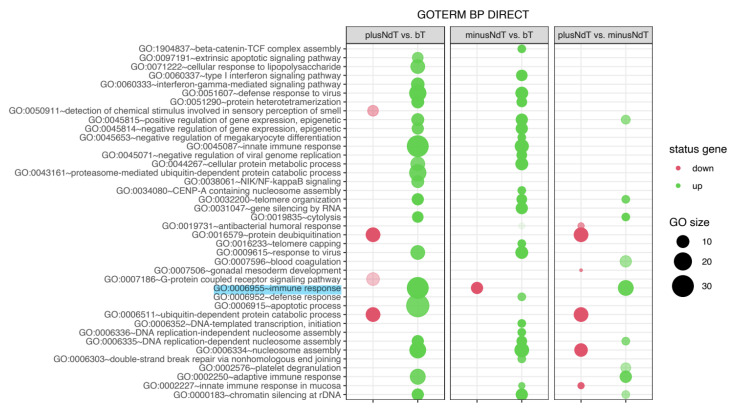
The bubble plot of biological processes assigned according to gene ontology (GO) classification. Genes assigned to individual processes fulfilling the criteria—adjusted *p* < 0.05, method of Benjamini, and minimum number of genes per group = 5—are presented. The bubble size indicates the number of genes represented in the corresponding annotation.

DAVID Bioinformatics Resource (Database for Annotation, Visualization, and Integrated Discovery) was used for functional annotation and enrichment analysis. This database is an integrated tool, biological knowledgebase, and tool to systematically extract substantial biological terms associated with a given gene list. The detailed DAVID analysis was performed as previously described [23,24]. The altered expression genes were classified into individual biological processes according to the Gene Ontology classification and presented in the bubble plot (Figure 2). The most significant changes pertained to the increased processes related to the immune response (GO:0006955) in patients with neuropathy during treatment. In addition, the neuropathy group showed an increase in processes such as GO:0006915~apoptotic process GO:0006334~nucleosome assembly and GO:0000183~chromatin silencing at rDNA and a reduction in processes such as GO:0016579~protein deubiquitination and GO:0006511~ubiquitin-dependent protein catabolic process, which may also contribute to the development of neuropathy.

#### 3.2.3. Cytoscape Analysis

Cytoscape is a software for visualizing complex networks and pathways. The use of this tool allows the presentation of the influence of individual genes on specific biological processes. The genes in the diagrams (Supplementary Figure S1–S3) marked in green represent upregulated expression in the neuropathy group. Graphic visualization of the genes identified in this study shows their significant involvement in the processes related to the immune response.

#### 3.2.4. Validation of Dysregulated mRNAs

**Figure 3:**
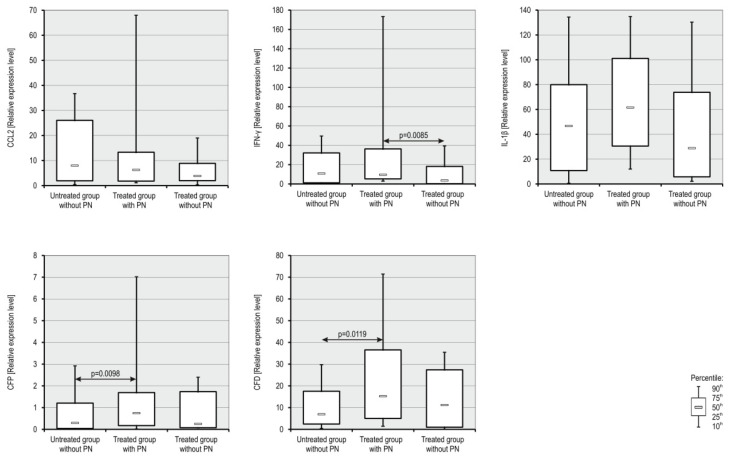
Box and whisker plot of gene expression levels in patients with MM. The boxes extend from the 25th percentile to the 75th percentile; the whiskers represent the 90th percentile and 10th percentile coefficients and the small black box indicates the expression median.

The results obtained from genetic microarrays were confirmed on selected genes involved in the immune response, on RNA samples from all patients by qRT-PCR. The results from the genetic arrays for selected genes (*CCL2*, *IFN-γ*, *IL-1β*, *CFP*, *CFD*) are presented in Supplementary Table S1. Selected genes showed increased expression in the neuropathy group compared with the expression in patients without neuropathy before and during treatment. Patients without symptoms of neuropathy before and during treatment showed similar gene expression levels (Table 2, Figure 3). A statistically significant increase in the expression of the gene coding IFN-γ was observed in PN patients during treatment compared with patients without neuropathy during treatment. Moreover, an interesting observation, though not statistically significant, was the increase in expression of the gene encoding IL-1β in PN patients during treatment compared with patients without PN before and during treatment (*p* = 0.0516). 

### 3.3. Concentration of Selected Proinflammatory Factors in the Plasma of Patients with MM

**Figure 4:**
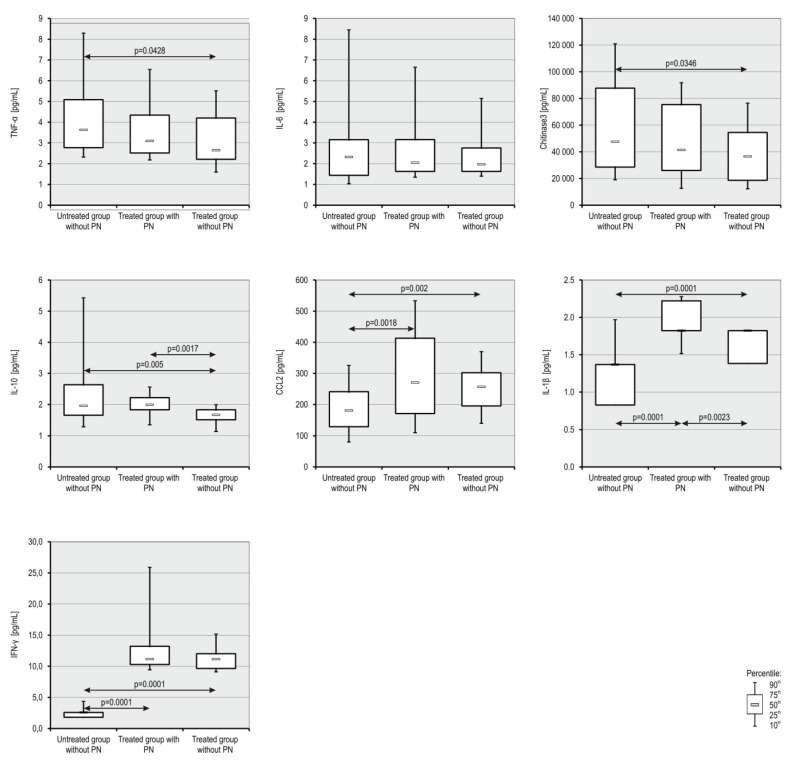
Box and whisker plot of concentrations of selected proinflammatory factors in patients with MM. The boxes extend from the 25th percentile to the 75th percentile; the whiskers represent the 90th percentile and 10th percentile coefficients and the small black box indicates the concentration median.

In Table 3 and Figure 4, we present the analysis of the concentration of selected proinflammatory factors in the plasma of patients from three groups using the Luminex method. The untreated group without neuropathy showed higher concentrations of TNF-α, Chitinase3, IL-6, and IL-10 than the other groups. In contrast, the concentrations of CCL2, IL-1β, and IFN-γ were significantly lower in the untreated group than in the other groups. The most important observation was the statistically significant increase in IL-1β in the neuropathy group compared with patients before and during treatment without neuropathy. In addition, we observed higher plasma concentrations of CCL2 in patients with grade 3 neuropathy (mean ± SD; 230.31 ± 97.80) compared with patients with grade 1 neuropathy (mean ± SD; 219.62 ± 109.44), though statistically insignificant. 

### 3.4. Correlations

In order to demonstrate the correlation of clinical parameters and the examined factors, a Spearman’s rank correlation coefficient (rs) analysis was performed. 

Supplementary Tables S2–S4 show the correlations between various parameters, including only those that showed a statistically significant result with at least one factor. Moreover, all the correlations described below were statistically significant (*p* < 0.05).

In the group with neuropathy during treatment, we observed interesting moderate-positive correlations between kappa FLCs and the expression of the *IFN-γ* gene (rs = 0.447) and between lambda FLCs and the concentration of CCL2 in the plasma (rs = 0.414). Expression of *IFN-γ* gene was moderately correlated with *CCL2* gene expression (rs = 0.645). The only negative correlation in the above group was demonstrated between the concentration of CCL2 and IL-1β in plasma (rs = −0.430). In the group without neuropathy during treatment, a moderate positive correlation was found between the concentration of CCL2 in the plasma and WBC (rs = 0.561). Another interesting observation was the positive correlation between the expression of couples of genes, *CCL2* and *IFN-γ* (rs = 0.564) and FactorD and IL-1β (rs = 0.514). 

## 4. Discussion

The direct cause of many neurological disorders still remains unknown. Various neurological diseases with heterogeneous pathogenesis are mediated by inflammatory reactions. Neurodegeneration is believed to be a consequence of an ongoing autoimmune process and/or related to increased inflammatory response [25]. Recent evidence suggests the impact of inflammatory responses on the development of Alzheimer’s disease, Parkinson’s disease, Huntington’s disease, amyotrophic lateral sclerosis, and stroke. The complement system, the synthesis of inflammatory mediators, and the recruitment of leukocytes play a pivotal role in the above processes [26].

The role of proinflammatory factors in the development of DiPN is mostly investigated in suboptimal in vitro studies and animal models. Therefore, in the present study, we decided to examine the most frequently analyzed inflammatory factors, such as TNF-α, IL-1β, CCL2, and IL-6 in the plasma of patients with MM.

TNF-α is mainly secreted by macrophages, monocytes, T cells, and mast cells. It regulates versatile processes such as survival, differentiation, proliferation, and cell apoptosis [27] (Supplementary Figure S3). However, inappropriate or excessive activation of TNF-α signaling has been associated with chronic inflammation leading to a variety of disorders. Increased expression of *TNF-α* is observed in autoimmunological diseases [28], carcinogenesis, cardiovascular system diseases [29], or nervous system diseases [30]. In the mouse model, the elevated expression of genes encoding TNF-α and TGF-β1 factors were demonstrated in the dorsal root ganglia (DRG) after induction of neuropathy with BTZ. In addition, treatment with anti-TNF-α and anti-IL-6 antibodies reduced the decrease in the amplitude of action potentials of the sensory nerves and the loss of myelinated and unmyelinated fibers [18].

In other studies, increased expression of *TNF-α* and the phosphorylated form of JNK1/2 in the DRG of rats with bortezomib-induced neuropathy have been observed. Furthermore, the elimination of the gene encoding the receptor for TNF-α (TNFR1 and TNFR2) inhibited the phosphorylation of JNK1/2 proteins, reducing mechanical allodynia [31,32,33]. In our study, we did not observe any increase in plasma TNF-α concentrations. However, we did show a slight increase in expression (fold = 1.73) of *TNF-α* at the transcript level using genetic microarrays in treated MM patients with PN compared with patients before and during the treatment without symptoms of neuropathy.

Contradictory results may be due to differences in the type of tissue in which TNF-α levels were tested. The concentration of TNF-α can vary considerably in the DRG of the rodent relative to its concentration in human plasma. No information was found in the available literature on TNF-α concentration in rodent plasma to compare results from the same tissue.

However, studies by Zhao et al. [17] showed that administration of BTZ induced the expression of TNF-α in the serum of MM patients and correlated with the severity of neuropathy. The study was conducted with a very small number of patients (35 patients divided into two groups: without neuropathy and with neuropathy during BTZ therapy). In our study, we did not observe an increase in TNF-α in the plasma of neuropathic patients. However, in this study, patients were treated with complex regimens such as VTD or VMP and not only BTZ, as in the abovementioned studies.

CCL2 chemokines play an important role in the pathogenesis of a wide variety of disease processes including vascular permeability, attraction of immune cells during metastasis, neurological disorders, autoimmune diseases, obesity, and atherosclerosis [34]. BTZ treatment increased the CCL2 factor in the DRG in rats, and anti-CCL2 administration inhibited BTZ-induced mechanical allodynia [35]. In our study, we observed an increased concentration of CCL2 in the plasma of neuropathic patients, which is consistent with these studies.

Additionally, in the same study, an increase in the expression of the AFT3 factor was demonstrated [35]. ATF3 is released under the influence of physiological stress in various tissues and is also a marker of regeneration after damage to the ganglion neurons of the dorsal root [36]. Surprisingly, in our study, we observed a slight increase in *ATF3* gene expression in patients with neuropathy compared with patients without neuropathy (fold = 1.41 vs. fold = 1.19). Moreover, we demonstrated using Spearman’s rank correlation coefficient analysis that the CCL2 factor correlates with lambda FLCs and the expression of *CCL2* gene correlates with the expression of *IFN-γ* gene (Supplementary Table S3), thus confirming the important role of the immune response to the development of PN. Obtained data for the group without neuropathy during treatment also provide convergent information on the role of immune response in the development of PN. The positive correlation (plasma CCL2 concentration vs. WBC (Supplementary Table S4)) is reflected by a decreased level of WBC (Table 1) and decreased expression of *CCL2* gene (Table 2) in patients without PN during treatment. The correlations of clinical data with the studied factors give an excellent effect of observing the development of the disorder through the analysis of basic blood count data. Recent reports indicate the involvement of anemia manifested by reduced iron concentration, reduction of hematocrit, or of red blood cells in the development of PN [37,38].

IL-1β is an important mediator of the inflammatory response and is involved in many cellular activities, including differentiation, cell proliferation, and apoptosis. Moreover, it induces the activity of cyclooxygenase-2, which regulates the production of prostaglandins in the central nervous system and contributes to hypersensitivity to inflammatory pain [39].

In the mouse model, increased expression of IL-1β in the spinal dorsal horn was proved. Additionally, elevated levels of IL-1β correlated with an increase in p38-MAPK, which regulates cell differentiation, apoptosis, and autophagy [33]. In this study, we observed increased expression of the MAP2K7 factor (Supplementary Figure S3), which is involved in the pathway promoting proapoptotic processes by activating genes such as *BAX* and *TP53* (Supplementary Figure S3). 

Moreover, inhibition of IL-1β secretion by an IL-1 receptor antagonist has been shown to attenuate the mechanical allodynia induced by BTZ [33]. In our studies, we observed a statistically significant increase in IL-1β in the plasma of patients with PN (Table 3) compared with other groups. The above observation testifies to the influence of inflammation in the development of PN and sets a search direction for a biomarker of neuropathy or target for treatment strategy. Focus on the search for the DiPN biomarker is pivotal because earlier PN identification could prevent side effects of the therapy and improve the patient’s quality of life. In our recent study, we identified increased expression of miR-22-3p in the plasma of multiple myeloma patients with DiPN, which could serve in the future as a potential early marker of PN [40].

Another important observation in our study was the increased concentration of IFN-γ in the plasma of patients with neuropathy. IFN regulates various biological processes in the cell, mainly by activating the JAK-STAT pathway (Supplementary Figure S2). Moreover, it stimulates macrophages, which, in turn, induce direct antibacterial and anticancer mechanisms. Additionally, it directs the growth, maturation, and differentiation of many cell types, enhances the activity of NK cells, and regulates B-cell function [41]. 

On the other hand, IFN-γ concentration increases in states of nervous system damage, often leading to disease progression such as multiple sclerosis. In the course of damage to the nervous system, IFN-γ activates other immune cells and MHC I and II induce a strong immune response [42]. A direct toxic effect of IFN-γ on nerve cells has been demonstrated. IFN-γ induces neuronal dysfunction that occurs as dendritic beads in cortical neurons and enhances glutamate neurotoxicity by alpha-amino-3-hydroxy-5-methyl-4-isoxazolpropionic receptors (AMPA). In effect, it causes increased Ca^2+^ and nitric oxide production, and then reduces ATP production, leading to the formation of dendritic beads [43]. IFN-γ also regulates the conversion of resting to active microglia, which underlies the development of neuropathic pain [44].

The complement system is part of the innate and adaptive immune system. It consists of many plasma proteins and membranes that support tissue homeostasis. Deregulation of complement components influences the development of some autoimmune neurological and neurodegenerative diseases [45].

Properdin, as well as Factor D, are the components of the complement system. They act as an activator of the complement system by stabilizing the alternative pathway convertase [46,47]. The alternative pathway of complement activation is associated with many autoimmune diseases, such as arthritis and age-related macular degeneration [48]. Levin et al. demonstrated in a mouse model an increase in proteins in the complementary system including properdin and factor D, both in the peripheral and central nervous system, during neuropathic pain [49]. In our study, we were the first to demonstrate an increase in the expression of genes regulating the complement system such as *CFP* encoding properdin and *CFD* encoding Factor D in patients with neuropathy. Preliminary studies in this area may lead to the identification of important factors of neuropathy and contribute to demonstrating the role of complement in the development of neuropathy in patients with MM.

In conclusion, the presented study highlights an important role of proinflammatory cytokines and complement proteins in the development of DiPN in patients with MM. 

The obtained results, ranging from clinical data, through the concentration of proinflammatory factors in plasma, gene expression, and genetic microarrays, convergently revealed an increased activity of the immune response in MM patients with PN during treatment. Moreover, these studies mark a new path in the pathogenesis of DiPN, demonstrating the previously unknown effect of complement components on its development. In the future, extended studies might presumably contribute to the development of DiPN biomarkers or new treatment strategies based on blocking the secretion of factors responsible for the development of DiPN. The desirable prevention of neuropathy would be a milestone in improving the quality of life of MM patients. 

## Figures and Tables

**Table 1:** Clinical characteristics of the study groups.

	Untreated Group without PN (*n* = 59)	Treated Group with PN (*n* = 34)	Treated Group without PN (*n* = 27)	*p*-Value * Treated Group with PN vs. Untreated Group without PN	*p*-Value * Treated Group without PN vs. Untreated Group without PN	*p*-Value * Treated Group with PN vs. Treated Group without PN
	Mean ± SD	Mean ± SD	Mean ±SD			
Patient’s age (years)	63.13 ± 9.74	65.73 ± 7.76	62.59 ± 9.74	0.2828	0.8412	0.2787
Sex (male/female)	29/30	22/12	19/8	0.1210		
White Blood Cells (×10^3^/µL)	7.25 ± 3.24	6.47 ± 2.57	5.33 ± 2.69	0.3129	**0.0007**	**0.0125**
Platelets (×10^3^/µL)	236.30 ± 97.22	215.11 ± 103.08	215.59 ± 97.66	0.2268	0.3520	0.8902
Erythrocytes (mln/mm^3^)	3.54 ± 0.70	3.73 ± 0.50	3.73 ± 0.54	0.4279	0.2584	0.9272
Hemoglobin (g/dL)	10.94 ± 2.19	11.68 ± 1.80	11.94 ± 1.23	0.1168	**0.0319**	0.5759
Hematocrit (%)	32.13 ± 6.27	35.10 ± 3.76	35.45 ± 3.72	0.1726	**0.0204**	0.5837
MCV (fL)	90.69 ± 4.46	94.35 ± 4.78	95.68 ± 8.17	**0.0392**	**0.0057**	0.9708
MPV (fL)	10.18 ± 0.93	10.68 ± 1.36	9.97 ± 0.97	0.3089	0.3521	0.0947
Total Ca^2+^ (mmol/L)	2.34 ± 0.38	2.28 ± 0.05	2.27 ± 0.23	0.6237	0.9101	0.5116
Total protein (g/dL)	8.21 ± 2.07	6.95 ± 1.74	6.72 ± 0.94	**0.0005**	**0.0006**	0.4899
IgG (g/L)	25.30 ± 25.97	16.35 ± 17.92	5.9 ± 3.84	0.4085	0.1860	0.1775
IgA (g/L)	8.72 ± 16.79	3.79 ± 10.85	0.29 ± 0.21	0.9382	0.3750	0.1349
IgM (g/L)	0.31 ± 0.34	0.38 ± 0.23	0.25 ± 0.22	**0.0335**	1.000	1
FLCs kappa (mg/dL)	210.90 ± 272.85	143.27 ± 261.15	143.02 ± 291.30	0.1611	0.2517	0.8084
FLCs lambda (mg/dL)	130.56 ± 284.7	27.02 ± 60.38	21.14 ± 37.21	0.8770	0.8643	0.7959

* Mann–Whitney test except sex (Pearson’s chi-squared test). In bold, *p*-values < 0.05.

**Table 2:** Relative quantification of gene expression normalized by the BMG gene in patients with MM.

	Untreated Group without PN (*n*=59)	Treated Group with PN (*n*=34)	Treated Group without PN (*n*=27)	*p*-Value * Treated Group with PN vs. Untreated Group without PN	*p*-Value * Treated Group without PN vs. Untreated Group without PN	*p*-Value * Treated Group with PN vs. Treated Group without PN
	Mean ± SD	Mean ± SD	Mean ±SD			
*CCL2*	14.86 ± 15.37	19.73 ± 36.06	13.09 ± 30.19	0.5549	0.1727	0.5516
*IFN-γ*	26.04 ± 49.66	61.57 ± 137.09	14.94 ± 24.37	0.1003	0.2621	**0.0085**
*IL-1β*	58.60 ± 56.64	88.72 ± 109.22	48.06 ± 54.62	0.1962	0.4049	0.0516
*CFP*	1.38 ± 4.03	4.93 ± 15.53	1.44 ± 3.68	**0.0098**	0.3331	0.1118
*CFD*	16.97 ± 35.33	36.28 ± 61.80	16.79 ± 21.30	**0.0119**	0.6924	0.0571

* Mann–Whitney test. In bold, *p*-values < 0.05.

**Table 3:** Plasma concentrations of selected proinflammatory factors in patients with MM.

	Untreated Group without PN (*n* = 59)	Treated Group with PN (*n* = 34)	Treated Group without PN (*n* = 27)	*p*-Value * Treated Group with PN vs. Untreated Group without PN	*p*-Value * Treated Group without PN vs. Untreated Group without PN	*p*-Value * Treated Group with PN vs. Treated Group without PN
	Mean ± SD	Mean ± SD	Mean ± SD			
TNF-α (pg/mL)	4.44 ± 2.71	3.84 ± 2.05	3.55 ± 2.32	0.3222	**0.0428**	0.2327
IL-6 (pg/mL)	4.48 ± 8.32	3.04 ± 2.73	2.77 ± 2.25	0.9300	0.9221	0.9593
Chitinase3 (pg/mL)	59,018 ± 39,791.33	50,706 ± 33,595.14	40,502 ± 27,212.7	0.4437	**0.0346**	0.2013
IL-10 (pg/mL)	3.44 ± 7.00	2.07 ± 0.83	1.64 ± 0.34	0.9554	**0.005**	**0.0017**
CCL2 (pg/mL)	190.94 ± 81.88	299.24 ± 165.30	252.41 ± 91.15	**0.0018**	**0.002**	0.4458
IL-1β (pg/mL)	1.28 ± 0.48	1.93 ± 0.35	1.70 ± 0.48	**<0.0001**	**<0.0001**	**0.0023**
IFN-γ (pg/mL)	2.79 ± 1.50	14.40 ± 8.03	11.76 ± 3.19	**<0.0001**	**<0.0001**	0.2659

* Mann–Whitney test. In bold, *p*-values < 0.05.

## Data Availability

The data presented in this study are available upon request from the corresponding author.

## Supplementary Materials

The following are available online at https://www.mdpi.com/article/10.3390/jcm10194584/s1. Table S1. The list of the most down- and upregulated genes in all groups. Table S2. Spearman’s correlation coefficients for clinical data and investigated factors in the pretreatment group without neuropathy. Table S3. Spearman’s correlation coefficients for clinical data and investigated factors in the group with neuropathy during treatment. Table S4. Spearman’s correlation coefficients for clinical data and investigated factors in the group without neuropathy during treatment. Figure S1. Relationships between proinflammatory factors. Figure S2. Cell signaling after IFN-γ receptor activation. Figure S3. Cell signaling pathways following TNF activation.
