# Supplementary material for: The Potential Role of Proinflammatory Cytokines and Complement Components in the Development of Drug-Induced Neuropathy in Patients with Multiple Myeloma
Source: J Clin Med. 2021 Oct 4;10(19):4584. doi: 10.3390/jcm10194584 (PMC8509696; doi:10.3390/jcm10194584)
Supplement: Supplementary file 1 [file jcm-10-04584-s001.zip › tab s1.pdf]

Table S1. The list of the selected down and up regulated genes in all groups.

Gene symbol	Gene name	Fold change	adj. p.val
**A. plusNdT vs. bT**			
ALAS2	5'−aminolevulinate synthase 2	11.61	<0.01
SNORA13	small nucleolar RNA, H/ACA box 13	10.36	<0.01
SNORA50C	small nucleolar RNA, H/ACA box 50C	10.06	<0.01
SNORD70	small nucleolar RNA, C/D box 70	9.08	<0.01
SNORA26	small nucleolar RNA, H/ACA box 26	8.93	<0.01
SNORA5A	small nucleolar RNA, H/ACA box 5A	8.38	<0.01
H2AC13	H2A clustered histone 13	8.03	<0.01
LOC105377884	uncharacterized LOC105377884	7.11	<0.01
SNORD121A	small nucleolar RNA, C/D box 121A	7.05	<0.01
SCARNA14	small Cajal body−specific RNA 14	6.26	<0.01
CFD	complement factor D	3.99	<0.01
CFP	complement factor properdin	3.12	<0.01
IFN-GAMMA	interferon gamma	2.3	<0.01
IL-1BETA	interleukin 1 beta	1.74	<0.01
CCL2	C-C motif chemokine ligand 2	1.41	<0.01
USP17L12	ubiquitin specific peptidase 17 like family member 12	−22.59	<0.01
USP17L17	ubiquitin specific peptidase 17 like family member 17	−24.04	<0.01
USP17L18	ubiquitin specific peptidase 17 like family member 18	−24.99	<0.01
USP17L20	ubiquitin specific peptidase 17 like family member 20	−25.35	<0.01
USP17L11	ubiquitin specific peptidase 17 like family member 11	−25.62	<0.01
USP17L25	ubiquitin specific peptidase 17 like family member 25	−25.71	<0.01
USP17L22	ubiquitin specific peptidase 17 like family member 22	−26.35	<0.01
USP17L6P	ubiquitin specific peptidase 17 like family member 6, pseudogene	−27.12	<0.01
USP17L5	ubiquitin specific peptidase 17 like family member 5	−30.68	<0.01
ZNF595	zinc finger protein 595	−46.52	<0.01
**B. minusNdT vs. bT**			
SERPING1	serpin family G member 1	5.45	<0.01
HP	haptoglobin	3.77	<0.01
SIGLEC14	sialic acid binding Ig like lectin 14	3.71	<0.01
CHI3L1	chitinase 3 like 1	3.63	<0.01
SCARNA5	small Cajal body−specific RNA 5	3.49	<0.01
IFI44	interferon induced protein 44	3.47	<0.01
IFI44L	interferon induced protein 44 like	3.41	<0.01
H2BC8	H2B clustered histone 8	3.26	<0.01
RSAD2	radical S−adenosyl methionine domain containing 2	3.19	<0.01
CRISP3	cysteine rich secretory protein 3	3.13	<0.01
TRGV3	T cell receptor gamma variable 3	−2.98	<0.01
CD22	CD22 molecule	−2.98	<0.01
CD79A	CD79a molecule	−3.23	<0.01
JCHAIN	joining chain of multimeric IgA and IgM	−3.29	<0.01
DEFA4	defensin alpha 4	−3.32	<0.01
IL18RAP	interleukin 18 receptor accessory protein	−3.39	<0.01
OLAH	oleoyl−ACP hydrolase	−3.98	<0.01
IL18R1	interleukin 18 receptor 1	−4.01	<0.01
PFKFB2	6−phosphofructo−2−kinase/fructose−2,6−biphosphatase 2	−4.97	<0.01
IL1R2	interleukin 1 receptor type 2	−5.76	<0.01
**C. plusNdT vs. minusNdT**			

ALAS2	5'−aminolevulinate synthase 2	18.01	<0.01
SNORA13	small nucleolar RNA, H/ACA box 13	9.77	<0.01
SNORD70	small nucleolar RNA, C/D box 70	7.69	<0.01
TRGV3	T cell receptor gamma variable 3	7.65	<0.01
SNORA50C	small nucleolar RNA, H/ACA box 50C	7.64	<0.01
SNORA26	small nucleolar RNA, H/ACA box 26	7.63	<0.01
SNORA5A	small nucleolar RNA, H/ACA box 5A	7.16	<0.01
SNORD111	small nucleolar RNA, C/D box 111	6.12	<0.01
RNU5D−1	RNA, U5D small nuclear 1	6.10	<0.01
SNORD121A	small nucleolar RNA, C/D box 121A	5.92	<0.01
CFD	complement factor D	3.05	<0.01
IFN-GAMMA	interferon gamma	1.9	<0.01
CFP	complement factor properdin	1.77	<0.01
IL-1BETA	interleukin 1 beta	1.26	0.06
CCL2	C-C motif chemokine ligand 2	1.13	0.3
USP17L12	ubiquitin specific peptidase 17 like family member 12	−12.59	<0.01
USP17L25	ubiquitin specific peptidase 17 like family member 25	−13.71	<0.01
USP17L20	ubiquitin specific peptidase 17 like family member 20	−13.79	<0.01
LINC00965	long intergenic non−protein coding RNA 965	−13.80	<0.01
USP17L18	ubiquitin specific peptidase 17 like family member 18	−13.89	<0.01
USP17L22	ubiquitin specific peptidase 17 like family member 22	−13.98	<0.01
USP17L11	ubiquitin specific peptidase 17 like family member 11	−13.99	<0.01
USP17L6P	ubiquitin specific peptidase 17 like family member 6, pseudogene	−14.80	<0.01
USP17L5	ubiquitin specific peptidase 17 like family member 5	−15.99	<0.01
ZNF595	zinc finger protein 595	−31.57	<0.01
