# Supplementary material for: The Potential Role of Proinflammatory Cytokines and Complement Components in the Development of Drug-Induced Neuropathy in Patients with Multiple Myeloma
Source: J Clin Med. 2021 Oct 4;10(19):4584. doi: 10.3390/jcm10194584 (PMC8509696; doi:10.3390/jcm10194584)
Supplement: Supplementary file 1 [file jcm-10-04584-s001.zip › tab s2.pdf]

Table S2. Spearman’s correlation coefficients for clinical data and investigated factors in the pre-treatment group without neuropathy.

	WBC	RBC	HGB	HTC	PLT	MPV	Total protein	IgG	IgA	IgM	FLCs kappa	FLCs lambda	CCL2	IL-1beta	IFN-gamma	ExCCL2	ExINF-gamma	ExIL1beta	ExProperdin
WBC	1,000	0,081	0,114	0,092	**0,331**	0,126	-0,139	0,039	-0,245	0,074	-0,103	0,008	0,028	0,194	0,024	-0,031	-0,129	-0,103	-0,002
RBC	0,081	1,000	**0,956**	**0,966**	**0,393**	0,061	-0,195	0,016	0,038	**0,380**	-0,137	-0,049	0,006	0,184	0,009	**-0,290**	-0,158	0,018	0,003
HGB	0,114	**0,956**	1,000	**0,979**	**0,393**	0,074	-0,203	0,052	0,048	**0,329**	-0,221	-0,027	0,004	0,217	0,032	**-0,263**	-0,122	0,015	0,007
HTC	0,092	**0,966**	**0,979**	1,000	**0,405**	0,102	-0,239	-0,013	0,058	**0,347**	-0,226	-0,011	0,030	0,250	0,024	-0,240	-0,117	-0,019	-0,026
PLT	**0,331**	**0,393**	**0,393**	**0,405**	1,000	**-0,306**	**-0,296**	-0,102	-0,250	**0,303**	-0,053	-0,106	0,155	0,229	-0,084	-0,250	-0,037	0,107	-0,047
MPV	0,126	0,061	0,074	0,102	**-0,306**	1,000	-0,157	-0,261	0,170	-0,112	-0,076	0,016	0,117	-0,052	0,147	0,026	-0,217	**-0,295**	-0,025
Total protein	-0,139	-0,195	-0,203	-0,239	**-0,296**	-0,157	1,000	**0,644**	0,093	-0,219	-0,074	-0,053	-0,012	0,163	0,138	0,131	0,231	0,101	0,208
IgG	0,039	0,016	0,052	-0,013	-0,102	-0,261	**0,644**	1,000	**-0,403**	0,164	-0,070	0,120	-0,151	0,222	0,121	0,120	0,021	0,061	0,012
IgA	-0,245	0,038	0,048	0,058	-0,250	0,170	0,093	**-0,403**	1,000	0,184	-0,198	0,155	-0,012	-0,055	0,130	-0,169	0,217	0,092	-0,049
IgM	0,074	**0,380**	**0,329**	**0,347**	**0,303**	-0,112	-0,219	0,164	0,184	1,000	-0,164	**0,391**	-0,052	0,013	-0,060	-0,264	-0,065	0,005	-0,077
FLCs kappa	-0,103	-0,137	-0,221	-0,226	-0,053	-0,076	-0,074	-0,070	-0,198	-0,164	1,000	**-0,478**	-0,085	-0,177	-0,110	-0,062	-0,126	**0,269**	-0,046
FLCs lambda	0,008	-0,049	-0,027	-0,011	-0,106	0,016	-0,053	0,120	0,155	**0,391**	**-0,478**	1,000	-0,207	-0,024	0,085	-0,043	0,133	**-0,271**	0,082
CCL2	0,028	0,006	0,004	0,030	0,155	0,117	-0,012	-0,151	-0,012	-0,052	-0,085	-0,207	1,000	**0,281**	**0,322**	0,082	0,035	0,165	0,063
IL-1beta	0,194	0,184	0,217	0,250	0,229	-0,052	0,163	0,222	-0,055	0,013	-0,177	-0,024	**0,281**	1,000	0,166	0,191	0,140	-0,035	0,141
IFN-gamma	0,024	0,009	0,032	0,024	-0,084	0,147	0,138	0,121	0,130	-0,060	-0,110	0,085	**0,322**	0,166	1,000	0,103	-0,079	0,129	-0,167
ExCCL2	-0,031	**-0,290**	**-0,263**	-0,240	-0,250	0,026	0,131	0,120	-0,169	-0,264	-0,062	-0,043	0,082	0,191	0,103	1,000	0,173	-0,241	0,113
ExINF-gamma	-0,129	-0,158	-0,122	-0,117	-0,037	-0,217	0,231	0,021	0,217	-0,065	-0,126	0,133	0,035	0,140	-0,079	0,173	1,000	-0,033	**0,265**
ExIL1beta	-0,103	0,018	0,015	-0,019	0,107	**-0,295**	0,101	0,061	0,092	0,005	**0,269**	**-0,271**	0,165	-0,035	0,129	-0,241	-0,033	1,000	-0,073
ExProperdin	-0,002	0,003	0,007	-0,026	-0,047	-0,025	0,208	0,012	-0,049	-0,077	-0,046	0,082	0,063	0,141	-0,167	0,113	**0,265**	-0,073	1,000

In bold, p-values <0.05
