# Supplementary material for: The Potential Role of Proinflammatory Cytokines and Complement Components in the Development of Drug-Induced Neuropathy in Patients with Multiple Myeloma
Source: J Clin Med. 2021 Oct 4;10(19):4584. doi: 10.3390/jcm10194584 (PMC8509696; doi:10.3390/jcm10194584)
Supplement: Supplementary file 1 [file jcm-10-04584-s001.zip › tab s3.pdf]

Table S3. Spearman's correlation coefficients for clinical data and investigated factors in the group with neuropathy during treatment.

	Age	RBC	HGB	HTC	MCV	PLT	MPV	Total protein	IgG	IgA	IgM	FLCs kappa	FLCs lambda	Degree of neuropathy	Treatment cycle	CCL2	IL-1beta	ExCCL2	ExINF-gamma	ExProperdin
Age	1,000	0,377	0,081	0,365	0,232	0,061	0,222	**-0,370**	-0,318	-0,284	0,136	-0,092	-0,247	-0,048	0,046	-0,083	0,040	-0,116	-0,077	-0,136
RBC	0,377	1,000	**0,915**	**0,988**	-0,444	0,018	0,231	-0,135	-0,086	1,000	1,000	**-0,733**	0,117	-0,060	-0,572	0,370	**-0,701**	0,139	0,152	-0,285
HGB	0,081	**0,915**	1,000	**0,952**	-0,280	-0,256	0,232	-0,123	-0,152	0,048	0,314	-0,102	-0,120	-0,044	-0,221	0,067	-0,300	0,080	0,040	-0,093
HTC	0,365	**0,988**	**0,952**	1,000	-0,426	-0,042	0,304	-0,055	-0,086	1,000	1,000	**-0,683**	-0,008	-0,121	-0,628	0,345	**-0,701**	0,176	0,236	-0,200
MCV	0,232	-0,444	-0,280	-0,426	1,000	-0,103	0,171	0,328	-0,086	-0,105	0,500	0,435	-0,172	0,151	0,238	**-0,681**	0,429	0,195	0,122	0,608
PLT	0,061	0,018	-0,256	-0,042	-0,103	1,000	**-0,825**	-0,081	0,246	-0,047	0,047	0,147	0,328	-0,064	0,153	0,332	-0,043	0,056	0,033	0,123
MPV	0,222	0,231	0,232	0,304	0,171	**-0,825**	1,000	0,119	0,571	-0,100	-0,200	-0,304	**-0,671**	-0,054	-0,536	**-0,642**	-0,068	-0,164	0,150	0,082
Total protein	**-0,370**	-0,135	-0,123	-0,055	0,328	-0,081	0,119	1,000	**0,602**	0,216	**-0,507**	**0,353**	0,091	0,071	-0,091	-0,162	0,293	-0,082	-0,043	0,074
IgG	-0,318	-0,086	-0,152	-0,086	-0,086	0,246	0,571	**0,602**	1,000	-0,171	-0,287	0,315	0,093	0,205	0,055	0,084	0,126	0,015	0,107	0,085
IgA	-0,284	1,000	0,048	1,000	-0,105	-0,047	-0,100	0,216	-0,171	1,000	-0,203	0,244	0,177	-0,176	-0,213	-0,325	0,045	-0,189	-0,079	**-0,425**
IgM	0,136	1,000	0,314	1,000	0,500	0,047	-0,200	**-0,507**	-0,287	-0,203	1,000	-0,327	0,184	0,084	0,053	0,093	0,210	0,153	0,008	0,322
FLCs kappa	-0,092	**-0,733**	-0,102	**-0,683**	0,435	0,147	-0,304	**0,353**	0,315	0,244	-0,327	1,000	-0,273	-0,251	0,008	-0,260	0,109	0,280	**0,447**	-0,069
FLCs lambda	-0,247	0,117	-0,120	-0,008	-0,172	0,328	**-0,671**	0,091	0,093	0,177	0,184	-0,273	1,000	**0,387**	-0,137	**0,414**	-0,002	-0,143	0,004	0,211
Degree of neuropathy	-0,048	-0,060	-0,044	-0,121	0,151	-0,064	-0,054	0,071	0,205	-0,176	0,084	-0,251	**0,387**	1,000	0,097	0,064	0,126	-0,121	0,042	0,032
Treatment cycle	0,046	-0,572	-0,221	-0,628	0,238	0,153	-0,536	-0,091	0,055	-0,213	0,053	0,008	-0,137	0,097	1,000	0,069	-0,248	**0,356**	0,208	0,036
CCL2	-0,083	0,370	0,067	0,345	**-0,681**	0,332	**-0,642**	-0,162	0,084	-0,325	0,093	-0,260	**0,414**	0,064	0,069	1,000	**-0,430**	-0,036	0,155	0,091
IL-1beta	0,040	**-0,701**	-0,300	**-0,701**	0,429	-0,043	-0,068	0,293	0,126	0,045	0,210	0,109	-0,002	0,126	-0,248	**-0,430**	1,000	-0,194	-0,156	0,096
ExCCL2	-0,116	0,139	0,080	0,176	0,195	0,056	-0,164	-0,082	0,015	-0,189	0,153	0,280	-0,143	-0,121	**0,356**	-0,036	-0,194	1,000	**0,645**	0,248
ExINF-gamma	-0,077	0,152	0,040	0,236	0,122	0,033	0,150	-0,043	0,107	-0,079	0,008	**0,447**	0,004	0,042	0,208	0,155	-0,156	**0,645**	1,000	0,173
ExProperdin	-0,136	-0,285	-0,093	-0,200	0,608	0,123	0,082	0,074	0,085	**-0,425**	0,322	-0,069	0,211	0,032	0,036	0,091	0,096	0,248	0,173	1,000

In bold, p-values <0.05
