# Supplementary material for: The Potential Role of Proinflammatory Cytokines and Complement Components in the Development of Drug-Induced Neuropathy in Patients with Multiple Myeloma
Source: J Clin Med. 2021 Oct 4;10(19):4584. doi: 10.3390/jcm10194584 (PMC8509696; doi:10.3390/jcm10194584)
Supplement: Supplementary file 1 [file jcm-10-04584-s001.zip › tab s4.pdf]

Table S4. Spearman's correlation coefficients for clinical data and investigated factors in the group without neuropathy during treatment.

	Age	WBC	RBC	HGB	HTC	MCV	PLT	MPV	Total protein	FLCs lambda	Total Ca^{2+}	CCL2	IFN-gamma	ExCCL2	ExINF-gamma	ExIL1beta	ExProperdin	ExFactorD
Age	1,000	-0,167	-0,014	-0,115	0,022	0,037	-0,124	0,125	**-0,408**	**0,475**	0,159	-0,230	0,091	0,070	0,072	-0,082	-0,211	0,226
WBC	-0,167	1,000	0,226	0,340	0,187	-0,258	**0,505**	-0,146	0,138	-0,137	0,268	**0,561**	-0,062	0,280	0,176	0,031	-0,018	0,185
RBC	-0,014	0,226	1,000	**0,828**	**0,895**	**-0,716**	0,386	-0,382	-0,205	-0,001	0,215	0,262	0,110	0,011	0,291	**-0,403**	0,199	-0,293
HGB	-0,115	0,340	**0,828**	1,000	**0,893**	**-0,417**	0,291	-0,283	0,017	0,038	0,248	0,180	-0,018	-0,023	0,274	-0,227	-0,010	-0,128
HTC	0,022	0,187	**0,895**	**0,893**	1,000	-0,371	0,261	-0,272	-0,180	0,128	0,290	0,160	-0,029	-0,020	0,356	-0,342	0,258	-0,292
MCV	0,037	-0,258	**-0,716**	**-0,417**	-0,371	1,000	**-0,444**	0,367	0,149	0,231	-0,135	-0,267	-0,228	-0,095	-0,002	0,363	0,021	0,045
PLT	-0,124	**0,505**	0,386	0,291	0,261	**-0,444**	1,000	**-0,427**	0,319	0,057	**0,483**	0,216	-0,127	0,168	0,247	-0,321	0,210	-0,209
MPV	0,125	-0,146	-0,382	-0,283	-0,272	0,367	**-0,427**	1,000	-0,322	-0,043	0,024	0,008	**-0,459**	-0,037	0,111	-0,005	-0,308	-0,021
Total protein	**-0,408**	0,138	-0,205	0,017	-0,180	0,149	0,319	-0,322	1,000	-0,065	0,152	-0,107	0,117	0,082	-0,020	-0,085	0,332	-0,153
FLCs lambda	**0,475**	-0,137	-0,001	0,038	0,128	0,231	0,057	-0,043	-0,065	1,000	-0,047	-0,315	-0,115	0,040	0,222	-0,070	-0,148	0,261
Total Ca^{2+}	0,159	0,268	0,215	0,248	0,290	-0,135	**0,483**	0,024	0,152	-0,047	1,000	-0,075	-0,343	0,100	0,141	**-0,621**	0,019	-0,405
CCL2	-0,230	**0,561**	0,262	0,180	0,160	-0,267	0,216	0,008	-0,107	-0,315	-0,075	1,000	0,212	0,153	0,179	0,083	-0,021	-0,054
IFN-gamma	0,091	-0,062	0,110	-0,018	-0,029	-0,228	-0,127	**-0,459**	0,117	-0,115	-0,343	0,212	1,000	0,058	-0,075	0,129	0,065	0,281
ExCCL2	0,070	0,280	0,011	-0,023	-0,020	-0,095	0,168	-0,037	0,082	0,040	0,100	0,153	0,058	1,000	**0,564**	-0,045	0,176	0,374
ExINF-gamma	0,072	0,176	0,291	0,274	0,356	-0,002	0,247	0,111	-0,020	0,222	0,141	0,179	-0,075	**0,564**	1,000	-0,069	0,186	0,022
ExIL1beta	-0,082	0,031	**-0,403**	-0,227	-0,342	0,363	-0,321	-0,005	-0,085	-0,070	**-0,621**	0,083	0,129	-0,045	-0,069	1,000	-0,166	**0,514**
ExProperdin	-0,211	-0,018	0,199	-0,010	0,258	0,021	0,210	-0,308	0,332	-0,148	0,019	-0,021	0,065	0,176	0,186	-0,166	1,000	**-0,471**
ExFactorD	0,226	0,185	-0,293	-0,128	-0,292	0,045	-0,209	-0,021	-0,153	0,261	-0,405	-0,054	0,281	0,374	0,022	**0,514**	**-0,471**	1,000

In bold, p-values <0.05
