# Supplementary figures and images for: The Potential Role of Proinflammatory Cytokines and Complement Components in the Development of Drug-Induced Neuropathy in Patients with Multiple Myeloma
Source: J Clin Med. 2021 Oct 4;10(19):4584. doi: 10.3390/jcm10194584 (PMC8509696; doi:10.3390/jcm10194584)

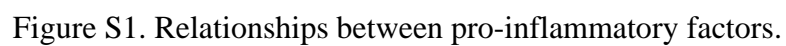

Figure S1. Relationships between pro-inflammatory factors.

Supplement: Supplementary file 1 [file jcm-10-04584-s001.zip › fig s1.pdf]

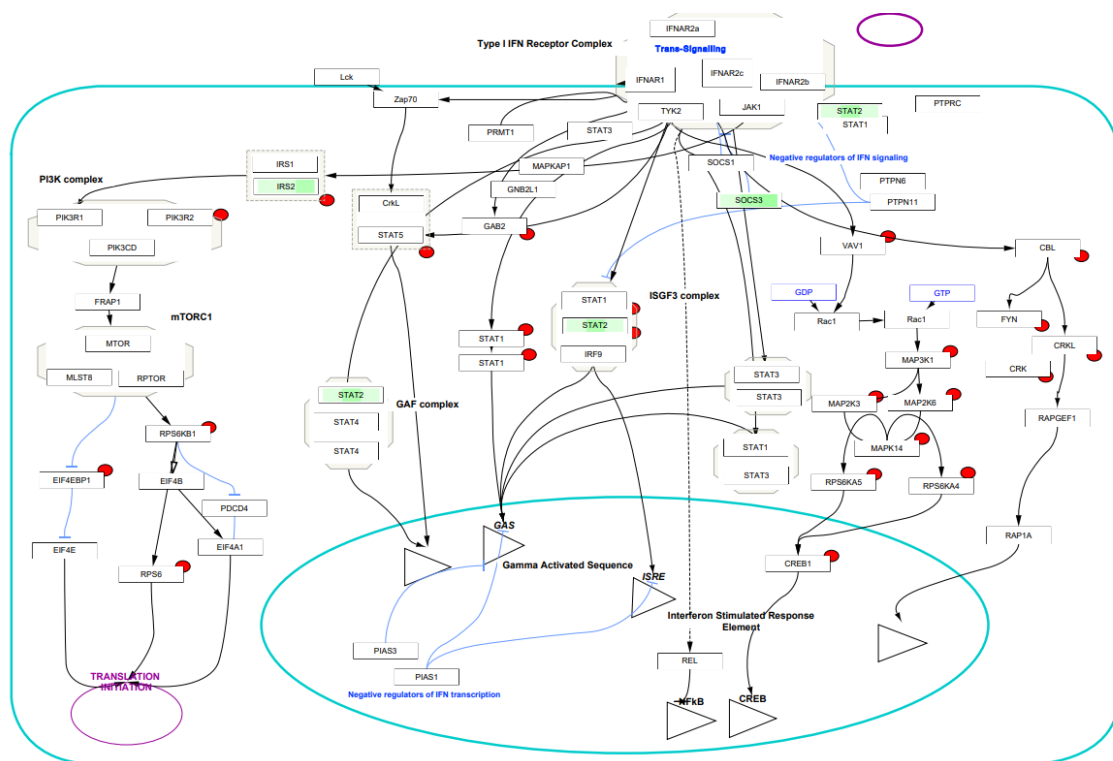

Figure S2. Cell signaling after IFN- $\gamma$  receptor activation.

Supplement: Supplementary file 1 [file jcm-10-04584-s001.zip › fig s2.pdf]

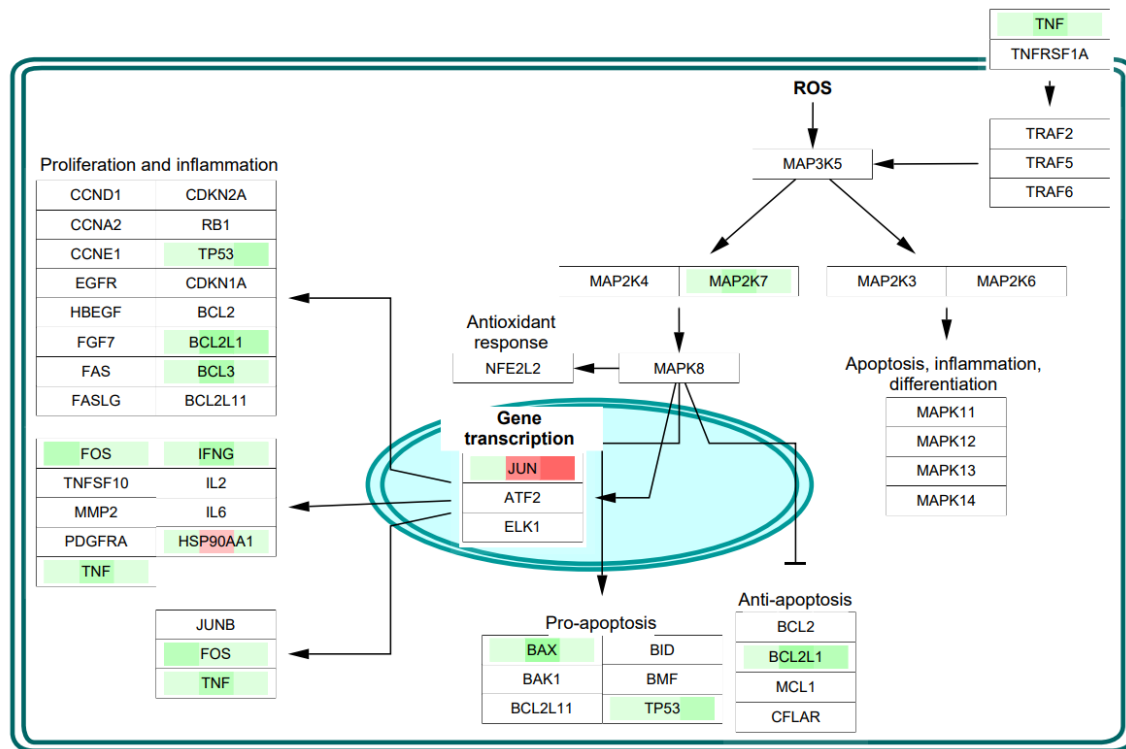

Figure S3. Cell signaling pathways following TNF activation.

Supplement: Supplementary file 1 [file jcm-10-04584-s001.zip › fig s3.pdf]
